# Supplementary material for: Patterns of Intron Gain and Loss in Fungi
Source: PLoS Biol. 2004 Nov 30;2(12):e422. doi: 10.1371/journal.pbio.0020422 (PMC532390; doi:10.1371/journal.pbio.0020422)
Supplement: Table S1 — Also available at http://genes.mit.edu/NielsenEtAl/. (4.3 MB ZIP). [file pbio.0020422.st001.zip › NielsenEtAl/html/855.html]

AN4987.1.NCU01166.1.MG07335.1.FG09908.1


```
 CLUSTAL W (1.82) Multiple Sequence Alignments - Introns Inserted


Sequence 1: NCU01166.1	385 aa
Sequence 2: MG07335.1	390 aa
Sequence 3: FG09908.1	399 aa
Sequence 4: AN4987.1	412 aa
Alignment Length: 427 aa
Number Identitical Residues: 194 aa
Alignment Score (without introns) 9148


MG07335.1 	MSASGFTSPFGANSNPFGSPEERRGVAGSIQPVLEEEE-ADGIGSEPISFK------SPG
NCU01166.1	MSSTGFTSPFG-NANPFGSS--GRSESGPMHRLVEEDE-NDTITSPTTPHFGVKNNAAPA
FG09908.1 	MSGP-FSSPFGENSNPFGTDR-GKRTNNAIHRVVEEEEENDTITSPTNARFGASPNSASM
AN4987.1  	MSNYSHSS-----NNPFLKVSTKEDKPSSFHKIAEDEE--YEVTSPTDATFRASKNADNS
          	**  ..:*      *** .    .   ..:: : *::*    : * . .    . .:   

MG07335.1 	FRAP-------------------------FGGG---DANSEGPPSSRPQN-PDG-YPAQY
NCU01166.1	FWNG-------------------------FGG----DAPSDMP--VRRQ--PDD-FPAHY
FG09908.1 	FSGP-------------------------FGGGFGGDSSAEAPSASRNVPNPDS-YPAQY
AN4987.1  	AGGNNPLESGRAGESGDGIRFGRDPFGNFQGGAEGQDESSIDPNGFRPTGGPDHGFPNNY
          	    .. .:. :..:...   . .. ..  **. . *  :  * . *   .** .:* :*

MG07335.1 	NFARRTSVSAESLKPIADSYDNWTPPFHPKSAEQLDRLKKAIQGNFLFSHLDDEQSAQIL
NCU01166.1	NLGRRTSVSAESLKPVTDNSDNWSPPVHPKTAEQLERLKKAISGNFLFNHLEDDQSAQVL
FG09908.1 	NFGRRTSVSAESLKPSADSYDNWSPPFHEKTPDQIERLKHAIEGNFLFSHLEDEQSAQIL
AN4987.1  	ALGRRTSVSAESLNPTSAGSDSWTPPSHPKSEEQLARLKTAVSNNFLFSHLDDEQSRTVL
          	 :.**********:* : . *.*:** * *: :*: *** *:..****.**:*:**  :*

MG07335.1 	GALVEKPIPAKDIK0VIVQGDAGDYFYVVEKGKFSVHVNSSGVMQAGTQGLGDHVGTIEA
NCU01166.1	GALVEKPVPAKGIK0VITQGDAGDYFYVVEKGRFEVYVNSTGALQPGPDGMGQKVGEIAE
FG09908.1 	GALVEKPIPAKGIK0VISQGDAGDYFYVVEKGSFDVYVNPSGSLQPGPDGMGNQVGNIQA
AN4987.1  	DALVEKPIPAKDIK~VISQGDAGDYFYIVEEGHFDVYINPSGSVQPGPDGAGTKISTIGP
          	.******:***.** ** *********:**:* *.*::*.:* :*.*.:* * ::. *  

MG07335.1 	GGSFGELALMYNAPRAATVMSAEPNCVLWALDRVTFRRILMESTFSRRRMYENFLEEVPI
NCU01166.1	GGSFGELALMYNAPRAATVVSAEPQCTLWALDRVTFRRILMESTFSRRRMYESFLEEVPI
FG09908.1 	GGSFGELALMYNAPRAATVISAEPGCTVWALDRVTFRRILMESTFARRRMYESFLEEVPL
AN4987.1  	GGSFGELALMYNAPRAATIVSTEPKSTLWALDRITFRRILMDSAFQRRRMYEAFLEEVPL
          	******************::*:** ..:*****:*******:*:* ****** ******:

MG07335.1 	LSTLTAYERSKIADALETQKYPPGTVVIKEGDPGEDFYLLECGEAEAFKAGIDQPVKLYK
NCU01166.1	LKTLTPYERSKIADALESQKYPAGHEIILEGDPGHSFFLLEAGEAAAFKRGNDSPVKNYK
FG09908.1 	LSTLNPYERSKIADALETQKFAPGEIIINEGDPGHAFYLLESGEADAYIGQPDNKVRHYK
AN4987.1  	LSSLKPYERAKIADALDTIKFPAGEYIIKEGDPGDAFYLLESGEAEAFMEGVEEPVKSYK
          	*.:*..***:******:: *:..*  :* *****. *:***.*** *:    :. *: **

MG07335.1 	KGDFFGELALLNDAPRAASVVSKTEVKVAALGKSAFQRLLGPVEPIMRRTRYDAIKTGVE
NCU01166.1	KGDFFGELALLNDAPRAASVISQTEVKVARLGKNAFQRLLGPIESILRRTRY----VEAE
FG09908.1 	KGDYFGELALLNDAPRAASIVAASPVKVGSLGKNAFQRLLGPVEGILRRTKYQGVATGVE
AN4987.1  	RGDYFGELALLDDKPRAASVRAKTEVKVAKLGRDGFKRLLGPVENIMRRTEY--------
          	:**:*******:* *****: : : ***. **:..*:*****:* *:***.*        

MG07335.1 	EMDPLQAA
NCU01166.1	EVDPLQVS
FG09908.1 	EMDPLHTG
AN4987.1  	SSRPSTAT
          	.  *  .
```
